# Control Over Believing and Doxastic Responsibility

**DOI:** 10.3389/fpsyg.2022.929143

**Published:** 2022-06-03

**Authors:** Aku Visala

**Affiliations:** Department of Systematic Theology, Faculty of Theology, University of Helsinki, Helsinki, Finland

**Keywords:** responsibility, belief, control, moral responsibility, doxastic responsibility

The theory of credition suggests that we should use active and agential language about beliefs. Instead of “beliefs” we should talk about “believing”. The approach encourages us to see believing as a “mental activity” or “dynamic activity” (Angel et al., 2017). These expressions raise an important question: is “believing” something that human agents do or does it happen to us? Or to put the question in another way: are our beliefs results of our own agency or not? In what follows, I will examine this question from the point of view of responsibility.

## Doxastic Responsibility and Control

For our responsibility attributions, it matters greatly if our beliefs can be said to be products of our agency. Consider the following argument:

(1) If epistemic responsibility attributions (e.g., blame and praise) are appropriate, we have voluntary control over our believing.
(2) We have no voluntary control over our believing.
(3) Therefore, epistemic responsibility attributions are not appropriate.

Premises (1) and (2) seem quite plausible. We humans assume that in order for responsibility attributions to be appropriate the target of those attributions must be under the control of the agent. We think it unjust to blame a person for an action that she did not control.

Furthermore, we often take beliefs to be analogous to actions; we treat beliefs as an expression of an agent's agency. If someone holds an irrational belief, we blame the person for failing to achieve expected epistemic standards. Notice, that this attribution of blame assumes that whether a person fulfills or fails those epistemic standards falls under the control of the person. Again, if the person exercised no control over her epistemic life, there would be no point in blaming her for the failure.

The second premise seems plausible as well. Choosing one's beliefs seems, after all, impossible. I cannot decide or choose to believe whether there is a computer is in front of me. If I see a computer before me, I believe it. If I do not see it, I do not believe it. We seem to be passive recipients of beliefs rather than authors of them. Our cognitive system produces beliefs without our conscious input on the basis of how it perceives the world. Since we do not choose our beliefs, we cannot be blamed or praised for them either.

Facing this dilemma, we have three options:

(a) Doxastic voluntarism.
(b)Doxastic involuntarism + ground epistemic responsibility judgments on something else than control over believing.
(c) Doxastic involuntarism + reject epistemic responsibility and revise our practices accordingly.

Most philosophers tend to gravitate toward options (b) and (c)^1^. Against this, I want to defend the plausibility of option (a), doxastic voluntarism. I will suggest that because philosophers have had such a high standard for what voluntary control requires, they have mistook believing as a passive process that does not involve the person's agency at all. We might not choose or decide to acquire most of our beliefs, but that does not mean believing is a passive process outside of our control.

## Two Arguments for Doxastic Involuntarism

Consider one practical and empirical argument against doxastic voluntarism. Robert Audi has argued that an evolved creature would be highly unlikely to develop a cognitive system that could acquire and maintain beliefs at will (Audi, 2015, p. 34–42). This is because holding a distinction between cognitive systems that represent the world and cognitive systems that facilitate and maintain the organism's aims and goals is crucial. If the organism fails to distinguish between how the world actually is and how the organism wants it to be, it will never achieve its goals. As a consequence, the processes of the “intellect” (getting at true beliefs) and the “will” (practical reasoning) will become independent over time.

More conceptual argument against doxastic voluntarism comes from Bernard Williams^2^. For Williams, beliefs are intrinsically aimed at truth. As such, they must be caused by something that is truth tracking or truth-apt, namely, something that makes a belief true (or false). This can be a piece of evidence, like perception, inference from knowledge, memory or something else. Consider now the possibility that I acquire a belief simply by forming an intention to acquire such a belief. If I know that I have acquired a belief simply because I have formed the intention to do so, I also know that this particular belief was not caused by a truth-tracking reason. Intentions to acquire a belief are not truth-tracking. So, if I know that I have decided to adopt a belief, I also know that the belief in question is not a product of a truth-tracking reason. This makes believing at will incoherent.

## Guidance Control

The two previous arguments strike true to me. It seems that synchronically deciding to believe something is psychologically very difficult and conceptually impossible. It does not follow from this, however, that we cannot exercise control over our beliefs. While we cannot synchronically choose or decide to believe something, there are accounts of control that can be applied to beliefs and have beliefs come out as free. The debate over action control in the literature on moral responsibility demonstrates that voluntary control can be much more varied and nuanced than simply consciously deciding to act just prior to action. Oftentimes, we exercise control over our actions diachronically, over time. A sufficiently deep account of control allows for control over beliefs as well without synchronic choice or decision in a way that still grounds attributions of epistemic responsibility.

In the debate about moral responsibility, many philosophers have argued that a person can be responsible for an action even in circumstances where the person does not have access to alternative possibilities. John Martin Fischer and Mark Ravizza, for instance, distinguish between what they call regulative control from guidance control (Fischer and Ravizza, 1998). When a person exercises regulative control over an action, the person has the ability to act or not to act. Fischer and Ravizza are convinced that Harry Frankfurt's counterarguments show how such control is not a necessary condition for moral responsibility. Whether blame or praise is appropriate is not determined by whether the agent had options, but rather the actual sequence of events that led to the action. Against this, guidance control is a form of control that requires no access to alternative possibilities (“choice”). Instead, a person exercises guidance control over an action when the sequence that leads to the action is a result of a mechanism that is both reasons-responsive and owned by the agent.

One philosopher that has applied the account of Fischer and Ravizza on believing is McCormick (2011, 2015). McCormick argues that reasons-responsiveness applies very well to mechanisms that produce beliefs. A useful test for responsiveness is to imagine various counterfactual scenarios. Perception, for instance, is quite responsive to reasons. Let us say I believe there is a computer in front of me, because there is a computer in front of me. Let us also imagine what would happen, if that computer were taken away. Most likely I would cease to believe that there is a computer in front of me. If my belief that there is computer in front of me were a product of a drug-induced delusion, for instance, it would not be so responsive to perceptual evidence. So, an actual sequence of events that includes my normally functioning perceptual system is reasons-responsive to a much higher degree than, say, an actual sequence that includes drug-induced hallucinations.

For Fischer and Ravizza, reasons-responsiveness is not enough for responsibility. A person cannot be said to appropriately control her actions, if those actions are not issued by a mechanism that does not properly belong to the agent. The agent must take responsibility for the outputs of those mechanisms and they must be her own. The previous example of drugs causing a change in one's perception is an example where the mechanism is not the agent's own. So, the challenge is to demonstrate how an agent could own and take responsibility over her belief-producing mechanisms. McCormick thinks that this challenge can be met.

## Taking Responsibility for One's Believing

Taking responsibility and owning one's belief-producing mechanisms are historical notions. First, I identify and recognize the kinds of tendencies my epistemic faculties have had and also begin to understand their consequences. Second, this diachronic process also extends to the future: I begin to accept that I am being blamed and praised on the grounds of how my epistemic mechanisms meet given standards. Fischer and Ravizza take this as a process of building up one's identity over time (Fischer and Ravizza, 1998, p. 210–217). We train children to respond to blame and praise until they eventually internalize most of the instruction. They begin to feel appropriate emotional responses and accept that they are judged on the basis of their behavior.

According to McCormick, a similar process of ownership can take place with respect to our epistemic faculties (McCormick, 2011). She takes one reactive emotion, guilt, as an example. She argues that we sometimes feel guilty for having a belief. If this is indeed appropriate, it reveals that we implicitly take beliefs to be a result of our agency. She also examines various belief-producing mechanisms, like perception and memory. Perception is a standard example of a mechanism with respect to which our agency is completely passive. McCormick points out that this is not so. There are epistemic standards against which we measure our management of our perception. We must be mindful of the circumstances and whether we are under the influence of perception-impairing chemicals, like drugs. Again, we can distinguish between those cases where a person's belief is a product of a sequence gone haywire (drugs or psychotic hallucinations, bad environment, etc.) and between normally functioning sequences. A failure to do so is a failure of accepted epistemic standards. When a person becomes a member of an epistemic community and internalizes its norms, she accepts that she can be blamed and praised according to how she manages to meet these standards. While perceptual systems are not under direct voluntary control—a person cannot decide to believe—she, nevertheless, exercises some control over maintenance of her perceptual systems. For failures of this maintenance, she can be held accountable.

## Conclusions

If the brief account I presented above is correct, it is indeed appropriate to describe and talk about believing as a dynamic process that involves our agency. On this account, believing does not simply happen to us but is a product of reason-responsive mechanisms that properly belong to us. Some of our beliefs are under our indirect control: we manage the cognitive mechanisms that issue them and control whether they are operate in the right environments. As members of an epistemic community, we have accepted that we are apt targets of epistemic blame and praise. If I manage my epistemic faculties poorly and adopt bad beliefs, I can be legitimately blamed for them.

## Author Contributions

The author confirms being the sole contributor of this work and has approved it for publication.

## Funding

This paper was funded by Dr. Rüdiger Seitz, via the Volkswagen Foundation, Siemens Healthineers, and the Betz Foundation.

## Conflict of Interest

The author declares that the research was conducted in the absence of any commercial or financial relationships that could be construed as a potential conflict of interest.

## Publisher's Note

All claims expressed in this article are solely those of the authors and do not necessarily represent those of their affiliated organizations, or those of the publisher, the editors and the reviewers. Any product that may be evaluated in this article, or claim that may be made by its manufacturer, is not guaranteed or endorsed by the publisher.

## Footnotes

^1^Peels (2013, 2017), for instance, rejects doxastic voluntarism but grounds epistemic responsibility practices on something close to believing. For (c), see Levy (2007). For important contributions to the debate, see Steup (2001). See also, Alston (2005), p. 58–81.

^2^For a version of Williams' argument, see Buckareff (2014).
